# Silk sericin alleviates aberrant photoperiod-induced alterations in testicular and adrenal steroidogenesis in adult mice

**DOI:** 10.1186/s12958-022-01032-y

**Published:** 2022-11-18

**Authors:** Eman Hassan, Shahinaz Magdy, Amany Attaallah, Eman Gaber, Omnia Mansour, Rehab A. Gomaa, Hala Odessy, Maria Augustyniak, Lamia M. El-Samad, Abeer El Wakil

**Affiliations:** 1 Department of Biological and Geological Sciences, Faculty of Education, Alexandria University, Alexandria, Egypt; 2 Department of Zoology, Faculty of Science, Damanhour University, Damanhour, Egypt; 3 Department of Zoology, Faculty of Science, Alexandria University, Alexandria, Egypt; 4 Institute of Biology, Biotechnology and Environmental Protection, Faculty of Natural Sciences, University of Silesia in Katowice, Bankowa 9, 40-007 Katowice, Poland

**Keywords:** Photoperiodism, Environmental stressor, Steroidogenesis, Adrenal gland, Testis

## Abstract

**Background:**

Steroidogenesis is a complex process of sequential enzymatic reactions affected by climate change. Animals respond to altered day length, the so-called photoperiod, with changes in physiology. The study aimed to an evaluation of sericin effect in alleviating steroidogenesis disorders induced by disturbed photoperiod in mice.

**Methods:**

The animals were randomly divided into three groups according to the lighting cycle: a control group with a standard 12_Light_:12_Dark_ cycle, a short-term photoperiod group with a 6_Light_:18_Dark_ cycle, and a long-term photoperiod group with an 18_Light_:6_Dark_ cycle. Both short and long-term groups were subdivided into two equal subgroups: The placebo and the sericin-treated subgroups received, for five weeks from prepubertal throughout adulthood, one intraperitoneal injection per week of the solvent and 1 g sericin/kg body weight, respectively.

**Results:**

Selected oxidative stress parameters and testicular and adrenal steroidogenic capacities of adult mice were measured. After five weeks, the placebo group with impaired photoperiod showed a decrease in the quality and quantity of sperm and a reduction in testosterone, corticosterone, aldosterone, total antioxidant capacity, xanthine oxidase, and melatonin. At the same time, in these groups, there was an increase in the level of aromatase, malondialdehyde, cholesterol, and steroidogenic factor-1 (SF-1) expression in the adrenal cortex and an enhancement in histological lesions. Mice receiving sericin had parameters similar to the control group.

**Conclusion:**

Our findings reveal that silk sericin can reduce the stress caused by photoperiod disorders regarding testicular function, sex hormone levels, and sperm quantity and quality. Thus, sericin is a biocompatible protein with a promising potential for its use in the case of organisms living under an abnormal photoperiod.

## Introduction

The ability of organisms to adapt to cyclically changing environmental conditions is a predictor of a species’ success and ensures survival. The length of the day, the so-called photoperiod, is undoubtedly the most reliable and predictable information that allows individuals to set the biological clock of a species and thus synchronize activities such as development, growth, migration or dormancy, reproduction, and many other vital functions. Therefore, photoperiodism is a firmly established mechanism evolved to optimize species’ physiological processes and regulate diurnal/seasonal activity for success [1, 2].

The relationship between photoperiod and the process of steroidogenesis and gametogenesis has been well described. The retina records the photons of light, and information is transferred to the suprachiasmatic nuclei (SCN) – a master biological clock coordinating all other clocks by influencing the pineal gland. The SCN regulates melatonin secretion and affects the hypothalamic-pituitary-gonadal (HPG) axis. Melatonin impacts gonadotropin-releasing/inhibitory hormone (GnRH; GnIH), which, together with kisspeptin (Kiss) and other neuropeptides, regulate gonadotropin production and secretion and consequently control gonadal development, steroidogenesis, and gametogenesis [3–8]. The adrenal glands, via the hypothalamus-pituitary-adrenal (HPA) axis, also receive information about photoperiod changes and secrete adrenal steroids, primarily cortisol and dehydroepiandrosterone sulfate (DHEAS) [9]. Studies in mice prove that light information is transmitted to the adrenal glands via the autonomic nervous system [10].

In mammals, the adrenal cortex and gonads share the exact developmental origin: the adreno-gonadal primordium (AGP). However, it involves complex molecular processes with many genes and networks acting synergistically or antagonistically. Wilms’ tumor suppressor gene 1 (WT1) is the gene responsible for the developmental process of AGP. This gene encodes a transcriptional regulator that plays a crucial role in forming many organs [11]. Indeed, WT1 and its target steroidogenic factor-1 (SF-1) are present early in the AGP development process. After adrenal gland and gonadal separation, WT1 is turned off within the adrenal primordium [12], whereas SF-1 persists in gonads and adrenal glands [13, 14].

However, these precisely controlled processes may be disturbed when incorrect/false information from the environment leads to a dysregulation of the master biological clock. Various pathological conditions are associated with inappropriate amounts of these hormones, including hormone-dependent tumors (prostate, breast, ovary), polycystic ovary syndrome, and autoimmune and inflammatory diseases [15, 16]. Besides its apparent benefits, the development of civilization has some negative consequences, including light pollution, which has been underestimated until recently. Exposure to artificial light at night (ALAN) causes disorganization of circadian rhythms resulting in sleep disorders, but also adversely affects metabolism, temperature regulation, immunological processes, and reproduction [17].

The diversity of mammalian animals is often faced on a seasonal basis with extra acclimatization to environmental stressors like temperature and the availability of food, water, and shelter. Studying photoperiodically sensitive animals in the laboratory differs according to various animals because it requires long-term changes as the animals can adapt to seasonal environmental variations. Moreover, critical photoperiodic changes between inhibitory and stimulatory periods are species-dependent [18, 19].

In this respect, the usage of natural products has increased nowadays, as they exhibit many biological benefits. Sericin is a natural macromolecular protein extracted from the silkworm cocoon *Bombyx mori* [20, 21]. It contains 18 amino acids, and the serine content is 32%. Serine and other hydrophilic amino acids exceeding over 42% make sericin water-soluble. During cocoon formation, sericin is secreted in the silk gland, and its primary role is cementing fibroin filaments, becoming the cocoon hard and stiff [22, 23]. Sericin has many pharmacological properties like antioxidant, anticoagulant, anti-cancer activities, cholesterol-lowering, and protection against ultraviolet-induced injuries that lead to apoptosis of keratinocytes [24].

Against this background, in the present study, we postulated that the binding effect of photoperiodic changes on mice might affect body weight and show changes in testes, adrenal structure, and physiology. The study aimed to check whether sericin can alleviate the induced changes. Thus, in the experiment, animals exposed to differential photoperiodism were treated with sericin. We hypothesized that such treatment might equilibrate the aberrant gonadal and adrenal structure, function, and steroidogenesis. For this purpose, the oxidative stress markers, including malondialdehyde (MDA), total antioxidant capacity (TAC), and xanthine oxidase (XO), as well as adrenal steroids, and the expression of SF-1 were evaluated in both the murine testis and adrenal glands after differential exposure conditions to photoperiodism associated with or without sericin treatment.

## Materials and methods

### Chemicals

Sericin powder from *Bombyx mori* (silkworm) was purchased from Sigma Aldrich, France (CAS Number 0474338686, Product Number S5201-5G). All reagents and chemicals were of analytical grade. Kits were purchased from Biodiagnostic and Research Reagents Co. (Cairo, Egypt).

### Animals

Twenty-five immature male albino mice (21 days old, 9–10 g) were used to investigate steroidogenesis, which is very sensitive to any alteration before puberty. After postnatal day 21, preputial separation (PPS) in males was assessed daily in mice. This consisted of attempts to retract the prepuce with gentle pressure manually. PPS is testosterone dependent and thus is an indicator of activation of the reproductive axis in males [25]. Puberty in rodents depends on weight [26]; hence, the weights of treated mice and control littermates were assessed in peripubertal mice through adulthood. The mice were obtained from the animal house of the Medical Research Institute, Alexandria University, Egypt. Mice were maintained following the recommendations of the Institutional Animal Care and Use Committee (IACUC), Alexandria University, under the supervision of the Ethical Committee of the University, and housed in clean cages with hardwood bedding. They had access to feed and clean tap water ad libitum. Animals were maintained in a controlled atmosphere at 23 ± 5 °C and 50–70% humidity and subjected to standard 12-h light and dark cycles.

### Experimental set-up

The animals were allowed to acclimatize to the laboratory conditions for one week before the experiment started. Then, they were randomly divided into three groups as follows:

1. Control group (CG, *n* = 5) in which mice were exposed to a standard lighting cycle that consisted of 12 h light and 12 h darkness from the beginning of the experiment and for five weeks;
2. Short-term photoperiod group (SPG, *n* = 10) in which mice were exposed to a lighting cycle of 6 h light and 18 h darkness from the beginning of the experiment and for 5 weeks. The mice in this group were subdivided according to the chosen treatment into 2 subgroups, five animals in each: the placebo subgroup (SPG-P) and the sericin-treated subgroup (SPG-S), receiving throughout the experiment a weekly intraperitoneal injection of normal saline and 1 g sericin/kg body weight, respectively.
3. Long-term photoperiod group (LPG, *n* = 10) in which mice were exposed to a lighting cycle that consisted of 18 h light and 6 h darkness from the beginning of the experiment and for 5 weeks. The mice in this group were subdivided into 2 subgroups, five mice in each according to the chosen treatment: The placebo subgroup (LPG-P) and the sericin-treated subgroup (LPG-S) receiving throughout the experiment one intraperitoneal injection of the solvent per week and 1 g sericin/kg body weight, respectively.

### Blood and organs collection

At the end of the experimental period, the animals were weighed and sacrificed by inhalation of isoflurane (2 mL/kg BW). Blood was collected by cardiac puncture and placed immediately on ice in test tubes. Serum was obtained from whole blood by centrifugation at 860×*g* for 20 min. The samples were kept at − 80 °C until further analysis.

### Tissue collection and preparation

Testes and adrenals were dissected, washed with chilled saline solution (0.9%), dried on tissue papers, and weighed. Then, the organs were divided as follows: the left side was immediately fixed in 4% paraformaldehyde and processed for routine histological or immunohistochemical analyses; the right side was used for biochemical analyses and the determination of oxidative parameters and hormonal assays. Tissues were minced and homogenized (10%, w/v) in ice-cold phosphate buffer (0.25 M, pH 7.4) in a Potter–Elvehjem type homogenizer. Homogenates were centrifuged at 10,000×*g* for 20 min at 4 °C to pellet cell debris. The supernatant was collected and stored at − 80 °C until the analyses were performed.

### Evaluation of epididymal sperm quality

The right epididymis was removed immediately, then sliced with a sharp razor blade in 5 mL Ham’s F12 medium and incubated for 5 min at 35 °C. Sperm were collected in the medium to detect associated abnormalities. Epididymal sperm quality analysis was conducted using computer-assisted semen analysis- Sperm VisionTM CASA System (MiniTUb, Tiefenbach, Germany) described by Krause [27].

### Biochemical analyses

#### Antioxidant enzymes assays

The level of the lipid peroxidation end product, malondialdehyde (MDA), was determined spectrophotometrically in the testes homogenates according to Tappel and Zalkin [28]. In the testes, the activity of total antioxidant capacity (TAC) was evaluated according to Koracevic et al. [29], and that of xanthine oxidase (XO) was assayed using the commercially available kit from PromoKine (Catalog number PK-CA577-K710). The measurement of XO was performed strictly according to the manufacturer’s instructions.

#### Steroids, hormonal assays, and aromatase estimation

Testosterone (serum and testicular tissue) (Catalog number ER1462, FineTest, Wuhan Fine Biotech Co., Ltd., Wuhan, Hubei, China), aldosterone (Catalog number KGE016, R&D Systems, Inc., USA), corticosterone (Catalog number ARG80652, Arigo Biolaboratories Corp., Taiwan), melatonin (Catalog number LS-F25805, LifeSpan BioSciences, Inc., USA), Dehydroepiandrosterone (DHEA) (Catalog number EU2945, FineTest, Wuhan Fine Biotech Co., Ltd., Wuhan, Hubei, China), and testicular aromatase (Catalog number RK03515, ABclonal, Woburn, MA, USA) levels were measured by specific immunoenzymatic assays.

### Histological and immunohistochemical analyses

The testes and adrenals were treated with a conventional grade of alcohol and xylol, embedded in paraffin, and then sectioned. Tissue sections (5–7 μm) were mounted on SuperFrost Plus slides (Menzel-Gläser, Thermo Scientific). Slides were deparaffinized in xylene, rehydrated through ethanol series, and stained with hematoxylin and eosin. For immunohistochemistry, epitope retrieval was achieved by 3 min boiling in a microwave in sodium citrate at 10 mM pH 6.0. After washing with PBS and blocking for 15 min with 5% bovine serum albumin in PBS with 0.1% Triton X-100, samples were incubated with a primary antibody against Sf-1 (Upstate Biotechnology Inc., Lake Placid, NY). The primary antibody was detected with the appropriate secondary antibody, coupled with horse radish peroxidase (HRP) (Santa Cruz, USA). HRP activity was detected with the chromogenic substrate ImmPACTDAB (SK-4105, Vector Labs, USA).

### Image analysis using computer-assisted assessment of quantitative immunohistochemistry

Slides were photographed using the Olympus® digital camera installed on the Olympus® microscope with ½ x photo adaptor, using 40x objective. The images were analyzed on Intel® Core I7® based computer using VideoTest Morphology® software (Russia) with a specific built-in routine for intensity measurement and object counting.

### Statistical procedures

A package of general linear models was used to determine the significance of differences in weight gain between the experimental groups. First, the data was transformed, and regression line equations were developed for all groups. The data was then analyzed using the equal/different slope model for validating the hypotheses of no or the presence of differences in body mass between groups. The Least Significant Difference test (ANOVA, LSD test, *p* < 0.05) was performed separately for body and testes weight and the ratio of testes weight/body weight at the fifth week of the experiment and for biochemical parameters. Before ANOVA analysis, the distribution of the data (using Kolmogorov-Smirnov and Lilliefors tests) and homogeneity of variance (applying the Levene test) were checked for all parameters. All measurements were performed in at least three replicates. The results were expressed as the mean ± SD in the figures. Principal Component Analysis (PCA) was performed separately for sperm parameters and biochemical parameters measured in the serum. The Pearson correlation coefficient was also calculated for selected parameters. Statistical analysis was performed using Statistica 13.1.

## Results

### Effects of sericin on photoperiod-induced testicular injury in mice

**Fig. 1:**
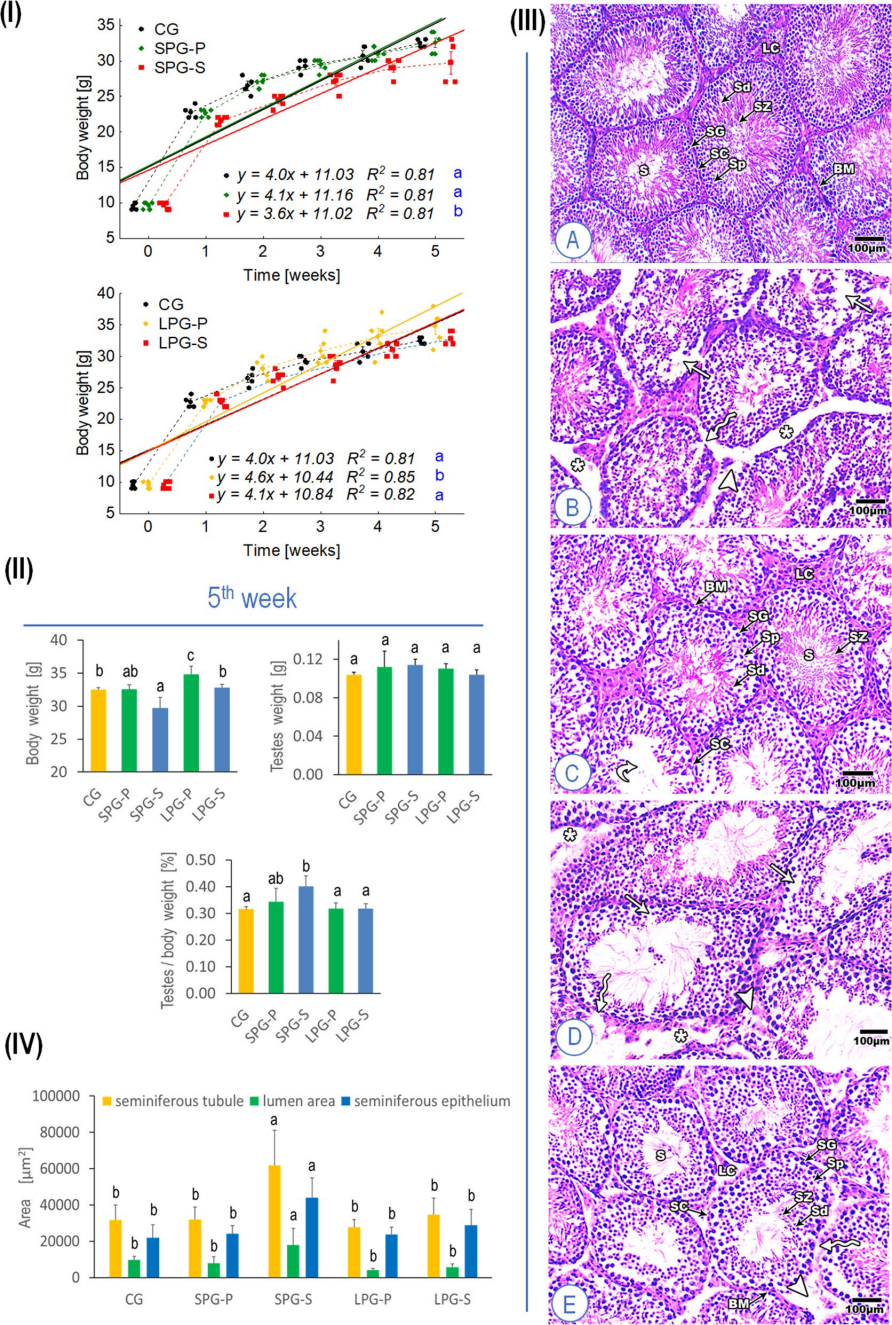
Effects of sericin on the testicular injury of mice induced by photoperiod. (**I**) The body weight of mice measured every three days. (**II**) Body weight in the fifth week, testes weight, and testes index expressed as the ratio of testicular weight to body weight. (**III**) Histopathology with H&E staining (200 and 400) of the testicular section on mice after treatment for five weeks. (**A**) Normal seminiferous tubule morphology, (**B**) affected testicular tissue after short photoperiod, (**C**) Sericin testicular tissue after short photoperiod showing normal seminiferous tubule morphology: few numbers of sperms in one seminiferous tubule, (**D**) Affected testicular tissue after long photoperiod, (**E**) Sericin testicular tissue after long photoperiod showing normal seminiferous tubule morphology. (**IV**) Morphometric analysis of cross-sectional area of seminiferous tubule, lumen area and seminiferous epithelium area Abbreviations: CG – control group, SPG-P – short photoperiod group placebo, SPG-S – short photoperiod group sericin-treated, LPG-P – long photoperiod group placebo, LPG-S – long photoperiod group sericin-treated. S – seminiferous tubules, BM – basement membrane, SG – spermatogonia, SP – spermatocytes, Sd – spermatids, Sz – spermatozoa, Sc – Sertoli cells, Lc – Leydig cells, * – enlarged intercellular spaces, normal arrow – degenerated seminiferous tubule, curved arrow – degenerate basement membrane, arrowhead – degenerate Leydig cells. The same letters denote homogenous groups (equal/different slope model; ANOVA, LSD test, *p* < 0.05)

The results revealed no significant differences in the body weights of SPG-P mice throughout the whole experiment duration as compared to the CG animals (Fig. 1I), while the body weights of SPG-S mice decreased significantly as compared to those at the CG and the SPG-P animals (equal/different slope model, *p* < 0.05). On the other hand, the weights of mice increased significantly under a long photoperiod exposure condition in the LPG-P animals compared to those from other groups. However, LPG-S mice showed a significant decrease versus LPG-P animals but not versus CG animals (equal/different slope model, *p* < 0.05) (Fig. 1I). As shown in Fig. 1II, there were no significant differences between testes weights in the different groups as well as testes index of mice except for the SPG-S animals which display a significant difference for the testes index (ANOVA, LSD test, *p* < 0.05).

Histological analysis of the H&E stained testis tissue sections showed the beneficial protective effect of sericin treatment against the damage caused to mice by differential photoperiodism exposure conditions. As compared to CG, SPG-P mice showed enlarged intercellular spaces, degenerated Leydig cells and basement membranes, atrophied seminiferous tubules with few Sertoli cells, spermatogonia, and primary spermatocytes (Fig. 1III and IV). Moreover, these observed alterations in the seminiferous tubules of the SPG-P animals became severely manifested in LPG-P mice. Interestingly, the treatment with sericin restored the normal histoarchitecture of the seminiferous tubules, the spermatocytes, and the number of Sertoli cells compared to those from differential photoperiodism exposure conditions alone (Fig. 1III and IV).

Morphometric measurements revealed that the cross-sectional area of the seminiferous tubules, lumen area, and seminiferous epithelium area is not significantly changed between different photoperiod exposure conditions compared to controls except for the SPG-S. Actually, there is an obvious significant increase in the lumen area as well as in the seminiferous epithelium area of SPG-S mice compared to CG animals (Fig. 1III and IV).

### Effects of sericin on sperm parameters of differential photoperiod exposed mice

#### Evaluation of epididymal sperm quality

**Fig. 2:**
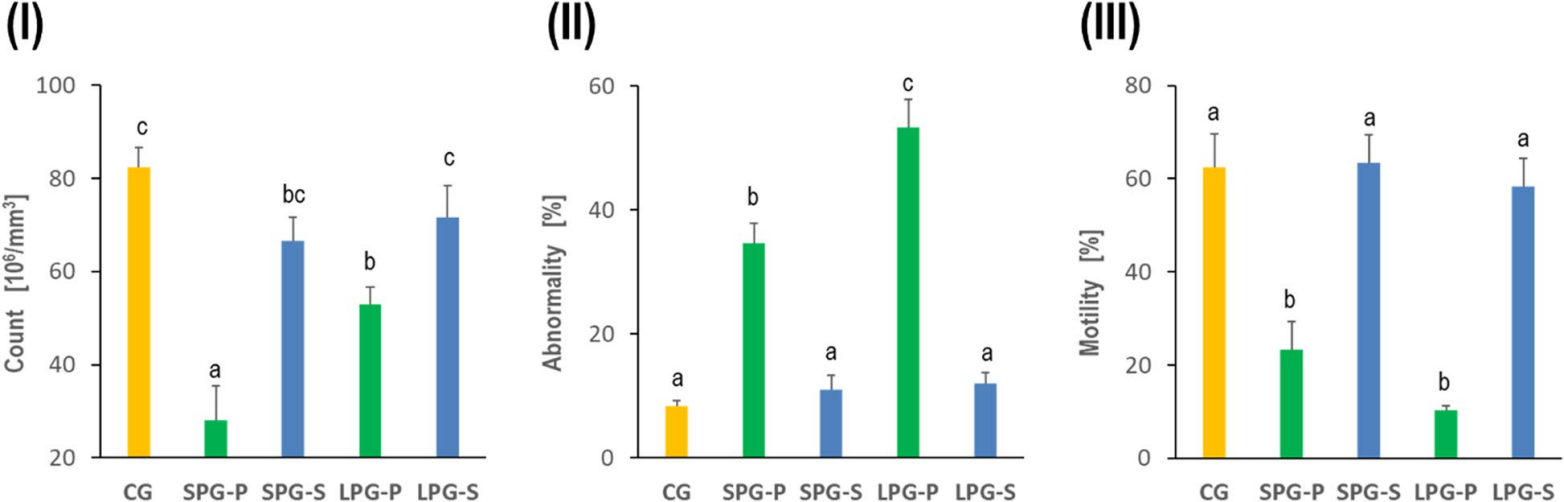
Effects of sericin on sperm quality and quantity of photoperiod-induced mice showing the sperm count (**I**), the number of abnormalities (**II**), and sperm motility (**III**). Abbreviations: as in Fig. 1

A significant decrease in sperm and an increase in the number of abnormal sperm has been recorded after five weeks of differential photoperiodism exposure. Moreover, the motility of the sperm was significantly reduced in animals kept under disturbed photoperiod. The combined treatment with sericin under altered photoperiod exposure conditions has significantly ameliorated the count and quality of sperm compared to non-treated mice. The use of sericin brought the discussed parameters to the value distinctive for the control group (Fig. 2).

### Effects of sericin on photoperiod-mediated alterations in reproductive hormonal levels

**Fig. 3:**
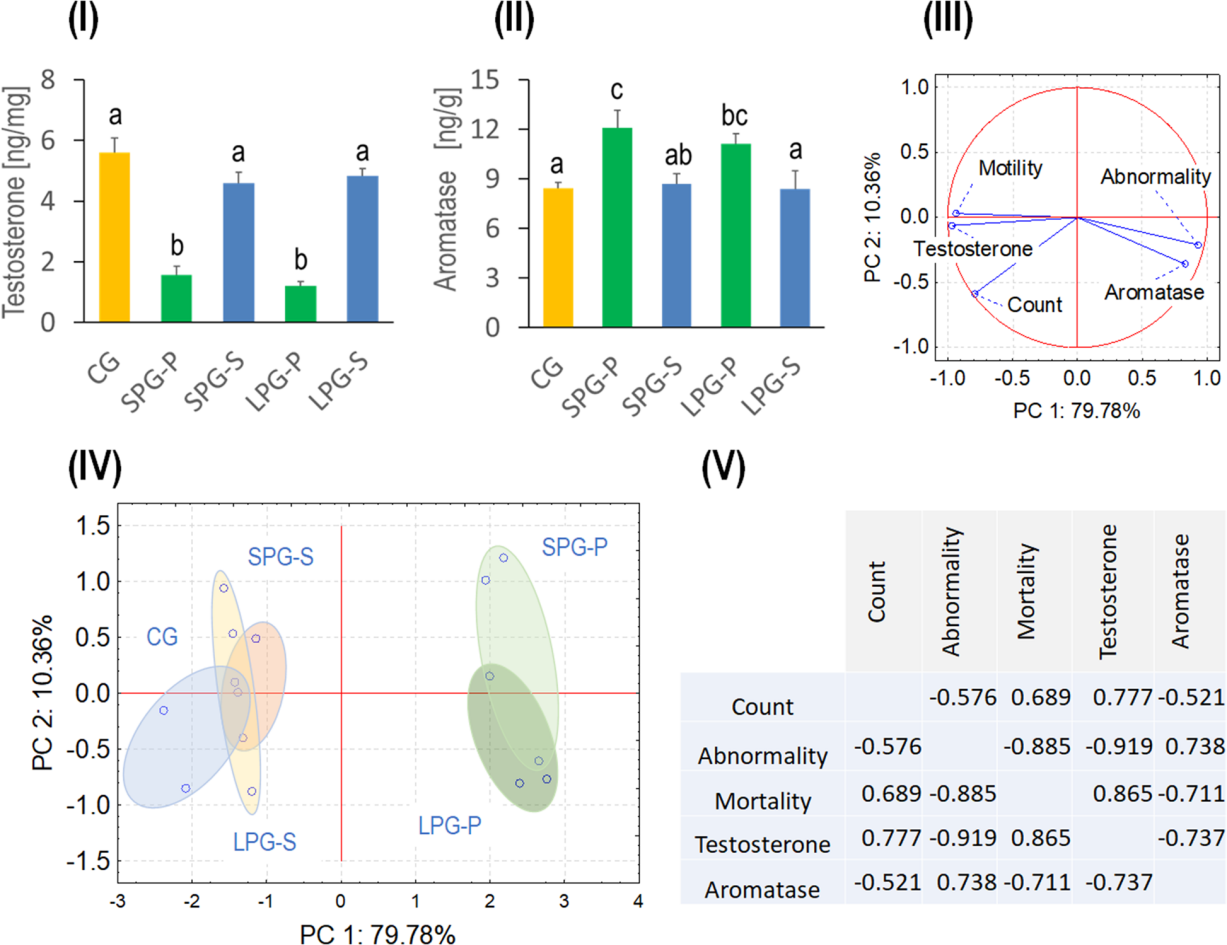
Effect of sericin on reproductive hormones of photoperiod-induced mice: (**I**) testosterone and (**II**) aromatase. (**III**-**IV**) Principal component analysis (PCA), illustrating relationships between sperm quality/quantity and tissue testosterone and aromatase levels. (**V**) Pearson correlation coefficients for sperm parameters and reproductive hormones. Abbreviations: as in Fig. 1

Male reproductive hormones regulate the process of spermatogenesis. As shown in Fig. 3I and II, differential photoperiod exposure has greatly affected mice’s hormonal secretion. The testosterone level decreased significantly in SPG-P and LPG-P mice compared to CG animals, while treatment with sericin restored the levels to the control level. The aromatase levels increased significantly in SPG-P and LPG-P mice compared to controls. However, the alteration was alleviated following sericin treatment, and the decrease was significant in SPG-S mice but not in LPG-S animals.

Sperm parameters, as well as hormone levels, were analyzed by PCA, and the variation of the data was reduced to two principal components. Principal component 1 (PC1) explained as much as 79.78% of the total variance, whereas principal component 2 (PC2) explained only 10.36% (Fig. 3III and IV). Sperm count was correlated with motility and testosterone level. The second tightly collated group was created by aromatase and abnormal sperm amount (Fig. 3III and V). 2D plot for all parameters revealed partly overlapped groups: CG, SPG-S, and LPG-S. The data for groups SPG-P and LPG-P partly overlapped but created an utterly distinct cluster from the control or the sericin-treated mice groups (Fig. 3IV).

### Effects of sericin on oxidative stress markers and xanthine oxidase level under differential photoperiod conditions

**Fig. 4:**
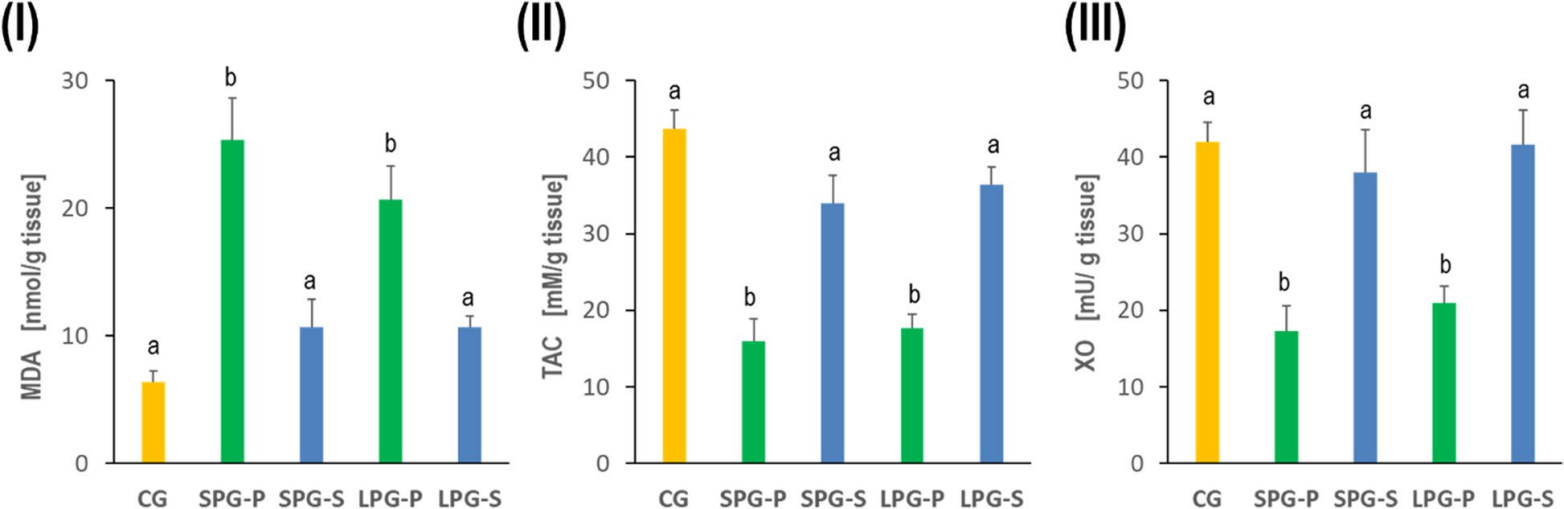
Effects of sericin on biomarkers of oxidative stress in testes tissues of mice induced by photoperiod: (**A**) malondialdehyde (MDA) level, (**B**) the total antioxidant capacity (TAC), and (**C**) Xanthine oxidase (XO). Abbreviations: as in Fig. 1

Oxidative stress due to differential photoperiodism exposure resulted in testis injury. In SPG-P and LPG-P mice, the lipid peroxidation product, MDA, augmented significantly (Fig. 4I), while the TAC and XO levels diminished significantly compared to CG mice (Fig. 4II and III). This alteration is reversible following sericin treatment, bringing the levels to the control levels.

### Effects of sericin on differential photoperiod-mediated adrenal histopathology

**Fig. 5:**
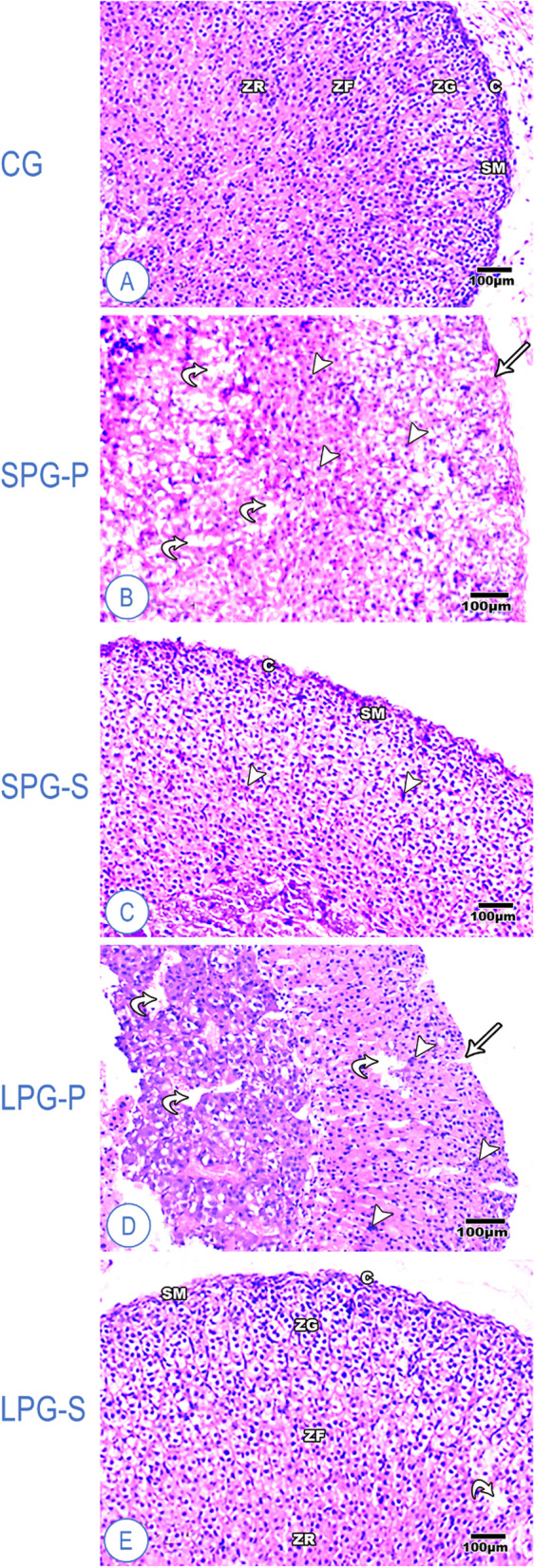
Representative histological sections of the adrenal cortex of mice (H&E). (**A**) Control group, showing a standard architecture of adrenal gland, (**B**) Short photoperiod group, showing mild affected adrenal gland, (**C**) Sericin-treated group after short photoperiod, showing the typical structure of adrenal gland (**D**) Long photoperiod group, (**E**) Sericin-treated group after long photoperiod showing a nearly typical structure of adrenal gland. Abbreviations: C – capsule of dense irregular connective tissue containing smooth muscles (SM); ZG – zona glomerulosa, ZF – zona fasciculata; ZR – zona reticularis, S – connective tissue septa, arrow – space in capsule cells, arrowhead – pyknotic nuclei, curved arrow – degenerate adrenal cells

The control adrenal gland showed a typical architecture comprising the cortex and the medulla. The gland is enclosed by a capsule of dense irregular connective tissue containing smooth muscles (Fig. 5). The adrenal cortex consists of the zona glomerulosa (ZG), zona fasciculata (ZF), and zona reticularis (ZR). The ZG is formed from oval or rounded clusters of cells with deeply stained nuclei. The cells of the ZF are radially organized into cords separated by capillaries. Some cells were large and polyhedral with pale vacuolated cytoplasm, while others looked less vacuolated with acidophilic cytoplasm and vesicular rounded nuclei. The ZR cells are small, deeply stained, and arranged in irregular cords and clusters separated by capillaries. Then, the adrenal medulla is located at the center of the gland. The cells were rounded in shape, slightly basophilic, and arranged in clusters (Fig. 5**A**). After differential photoperiodism exposure, the cytoarchitecture of the adrenal ZG and ZF in SPG-P and LPG-P showed aberrant organization and lost the capsule boundaries (Fig. 5**B** and **D**). Moreover, some cortical cells were shrunken, and their nuclei became pyknotic, while the medullary cells exhibited slight degenerative signs. The observed deterioration is more evident in LPG-P mice with highly congested blood capillaries throughout the different cortical layers (Fig. 5**D**). Silk sericin treatment improved the smooth muscles of the capsule that enveloped the adrenal layers, and many ZF cells appeared like normal ones, while some ZF and ZR cells were shrunken with hyper acidophilic cytoplasm and pyknotic nuclei (Fig. 5**C** and **E**).

### Effects of sericin on steroids and hormonal levels, as well as melatonin concentration under differential photoperiodism

**Fig. 6:**
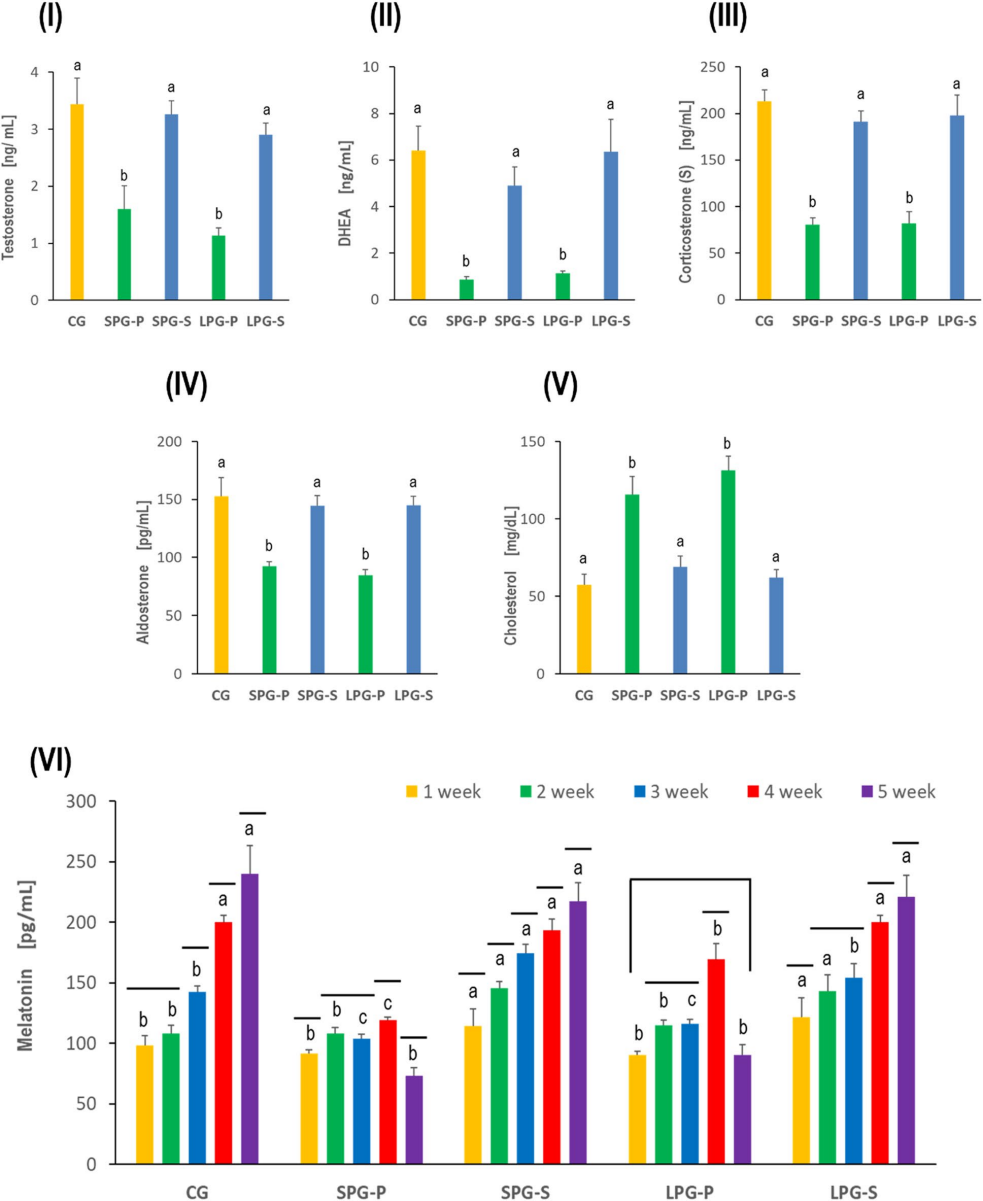
Effect of sericin treatment of short and long photoperiod on the serum level of (**I**) testosterone, (**II**) dehydroepiandrosterone DHEA, (**III**) Corticosterone, (**IV**) Aldosterone, (**V**) Cholesterol, and (**VI**) Melatonin. Abbreviations: as in Fig. 1. Concerning Melatonin (VI) - the same letters denote homogenous groups when comparing experimental groups in a given week. A horizontal line above the bars means a homogenous group when comparing melatonin concentration obtained in subsequent weeks in a given experimental group (ANOVA, LSD test, p < 0.05)

The levels of testosterone, DHEA, corticosterone, and aldosterone in the serum of mice exposed to different photoperiods were significantly decreased compared to controls. However, treatment with sericin in SPG-S and LPG-S mice restored the levels of the steroid hormones to the control levels and increased significantly as compared to SPG-P and LPG-P animals. On the other hand, cholesterol levels in the serum of SPG-P and LPG-P mice were significantly increased compared to CG animals. Interestingly, treatment with sericin significantly decreased the levels in SPG-S and LPG-S compared to SPG-P and LPG-P animals (Fig. 6).

Melatonin concentration increased in the following weeks of the experiment in animals from the control group and groups with altered photoperiod but treated with sericin (SPG-S and LPG-S groups). Mice from the placebo groups subjected to short or long photoperiod had significantly lower serum melatonin concentrations than the control and the sericin-treated groups (Fig. 6 VI).

### SF-1 expression fluctuations following differential photoperiodism exposure conditions

**Fig. 7:**
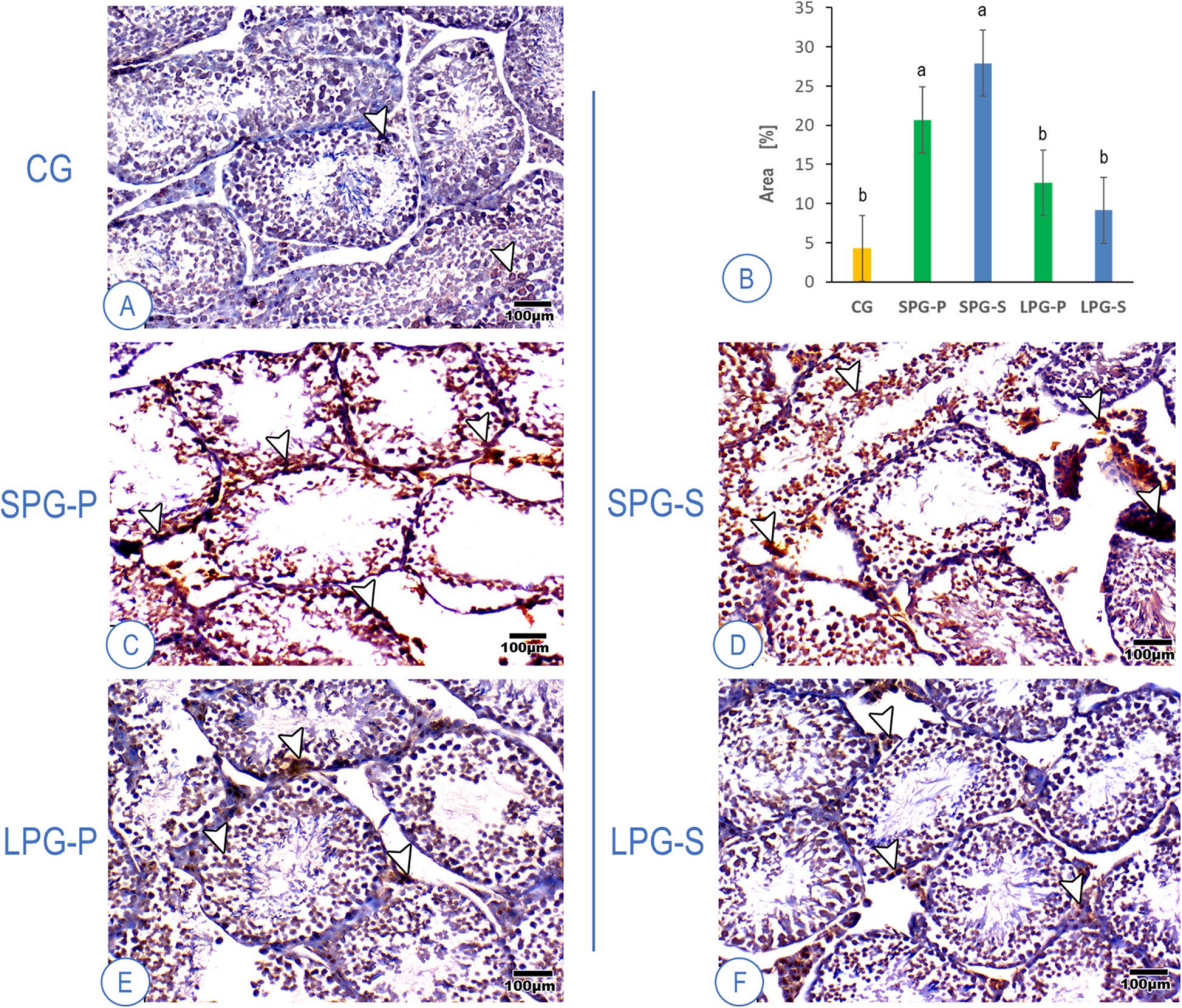
Mice testes labeled for SF-1 in the control group (**A**), showing standard expression; The percentage of positively stained cells in the experimental groups (**B**); Expression along the testes in the placebo groups (**C** and **E**); Expression in the sericin-treated groups (**D** and **F**), arrowhead – immunopositive cells among seminiferous tubules

**Fig. 8:**
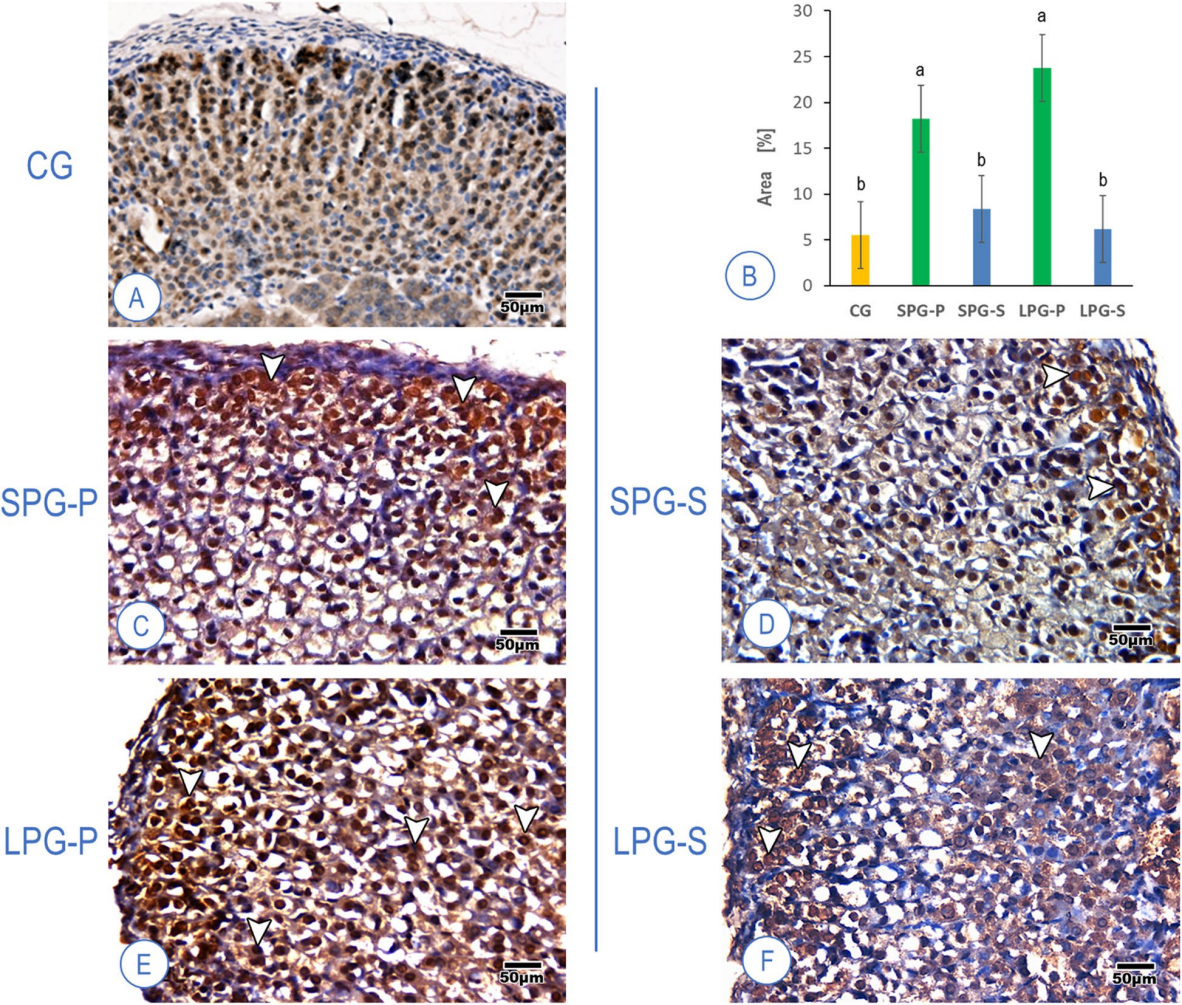
Mice adrenal cortex labeled for SF-1 in the control group (**A**), showing standard expression; The percentage of positively stained cells in the experimental groups (**B**); High expression along the adrenal cortex in the placebo groups (**C** and **E**); Moderate expression in the sericin-treated groups (**D** and **F**), arrowhead – immunopositive cells among ZG

Immunohistochemically stained slides were analyzed on the standard semi-quantitative basis incorporating the intensity of the staining (mild, moderate, strong) coupled with the percentage of positively stained nuclei (by counting cells) on a four-point scale: 0, no stain (up to 10% positive cells); 1, light (11 to 25% positive cells); 2, moderate (26 to 50% positive cells); 3, heavy (51 to 75% positive cells) and; 4, intense stain (76 to 100% positive cells). The cells were considered positive when more than 10% were stained with the respective antibodies (Figs. 7 and 8). The number of positively stained SF-1 cells in the testes increased in the short photoperiod groups, both in the placebo and sericin groups. Long photoperiod mice had SF-1 expression at the control level (Fig. 7). In the adrenal cortex, positively stained SF-1 cell numbers were increased in groups with altered photoperiod. The application of sericin attenuated this effect (Fig. 8).

## Discussion

Steroidogenesis is a multistep and dynamic process starting from the peripubertal stages throughout adulthood. The primary organs concerned by steroidogenesis, namely the testes and the adrenal glands, are susceptible to any alteration in this process prior to the completion of puberty [30]. This study aimed to investigate the beneficial impact of naturally-derived silk sericin upon testicular and adrenal steroidogenesis in mice under altered photoperiodism, starting with animals at the peripubertal stages and continuing throughout adulthood. In this context, we followed the previously reported working definitions of ages at stages of postnatal development, according to concentrations of gonadal hormones and performance of social behaviors [31].

Various studies report that disruption of photoperiod affects animal body weight, although the effects may differ among vertebrate groups. It was shown that the field vole (*Microtus agrestis*) transferred to long-day photoperiod (LD, 16_Light_: 8_Dark_) for four weeks increased its body weight by 24.8% compared to animals kept under short photoperiod conditions (SD; 8_Light_: 16_Dark_). Interestingly, enlarged testes and seminal vesicles were also found [32]. Also, rats subjected to LD photoperiod increased their body weight compared to animals exposed to SD photoperiod. However, Syrian hamsters react to such conditions in an opposite manner [33, 34]. Similar effects are caused by ALAN, leading to weight gain and obesity in humans, mice, and Australian budgerigars but not in toads *Rhinella marina* [35, 36]. In agreement with cited studies, in our experiment, LD photoperiod caused a significant increase in the weight of the mice after five weeks. Although the disturbance of the photoperiod did not affect the mass of the testicles, it led to visible histological changes (Fig. 1). This result is consistent with those described by Gouda and Selim [37] and Kus et al. [38]. They demonstrated that exposure to different duration of light has unfavorable effects on the testicular structure in rats. Interestingly, in our experiment, the treatment with sericin restored the typical structure of the seminiferous tubule, the layer of the spermatocytes, the number of Sertoli cells, and the standard structure of Leydig cells compared to disturbed photoperiod groups (Fig. 1).

Reproductive disorders due to circadian rhythm disruption have been less frequently studied in males/men than in females/women. Although it is known that the desynchronization of endogenous circadian clocks affect each level of the HPG axis, the level of sex hormones, the process of spermatogenesis, final sperm concentration and motility, and, ultimately, fertility - both in males of various vertebrate species and in humans [8, 39, 40]. It has been shown that mice exposed to shortened light-dark cycle (Light/Dark = 4 hours/4 hours) for 5 or 10 weeks responded with reduced testes size, abnormal morphology, decreased sperm concentration and motility, and lowered levels of dihydrotestosterone and androstenedione [41]. In our experiment, we found that 5-week exposure to both SD and LD photoperiods reduced sperm count and motility and increased the number of abnormal sperm (Fig. 2). These changes strongly correlated with decreased testosterone levels and, at the same time, increased aromatase levels in this gland (Fig. 3).

Aromatase belongs to the cytochrome P450 family and catalyzes the aromatization of androgens and their conversion to estrogens. The changes in aromatase concentration are season- and age-related. Aromatase is detected in the testicle (Leydig cells or Sertoli cells - depending on age, as well germ cells at each step of spermatogenesis, from gonocytes to spermatozoa). Aromatase is also present in the prostate, as well as also in the brain, the ovaries of females, and cancer tissues [42, 43]. For example, aromatase expression in bank vole testes was more remarkable in animals kept under long photoperiod in which spermatogenesis was fully developed. Thus, its role is substantial and is related to sperm motility properties and its indirect role in the regulation of spermatogenesis [43–46]. Again, sericin restored testosterone and aromatase levels to levels typical of control animals in our experiment.

The disturbance of the photoperiod also caused an increase in MDA and a decrease in TAC concentration, and a reduction of XO activity (Fig. 4). Such a result indicates that increased oxidative stress is an accompanying phenomenon and probably can contribute to the development of reproductive disorders. To link the effects of photoperiod with disorders of spermatogenesis and oxidative stress, it is worth paying attention to the vital hormone produced by the pineal gland, namely melatonin (Mel). This compound coordinates the master clock and regulates the circadian rhythm and, thus, physiological functions. However, Mel synthesis also occurs in other tissues/organs (e.g., digestive tract, platelets, skin, or bone marrow). Mel exhibits anti-inflammatory and antioxidant activity and is perceived as a reactive oxygen species (ROS) scavenger. Consequently, Mel can protect cells and tissues from infection and oxidative stress [47–49]. Rossi et al. [47], who examined men with idiopathic infertility, have clearly proven and transparently presented this relationship. Moreover, male Syrian hamsters and various cell lines showed a negative correlation between Mel concentration in the testes and the expression of proinflammatory factors (TNFα, IL1β, and COX2) as well as a positive correlation between Mel concentration and the expression of antioxidant enzymes such as catalase, superoxide dismutase, and peroxiredoxin. In our experiment, we confirmed that the inappropriate photoperiod in SPG-P and LPG-P groups disturbed the synthesis of Mel, which contributed to the intensification of oxidative stress (and perhaps also proinflammatory effects). The consequence was unfavorable changes in the weight and structure of the gonads and adrenal glands, sperm quantity and quality, hormone concentration, markers of oxidative stress, and cholesterol in the testes and/or serum (Figs. 1-8). Interestingly, such effects were observed not only when the day was extended (LPG-P) but also when it was shortened (SPG-P). This result suggests an even more complex nature of the phenomenon under consideration and encourages further research.

Using natural plant-derived compounds to reduce testicular toxicity caused by various factors is of interest to scientists. Mansour et al. [50] investigated the effects of *Ginkgo biloba* extract (EGb 761) in reducing methotrexate-induced adverse effects in rat testes. Methotrexate (MTX) is an anti-cancer and immunosuppressant drug with testicular toxicity and infertility side effects. Oxidative stress is generally believed to be involved in the toxicity of MTX. The four-week exposure of rats to MTX led to the development of a complete picture of testicular damage and disorders of spermatogenesis. It was manifested by organ fibrosis and a decrease in the quantity and quality of sperm, as well as in reduction in the level of FSH, LH, testosterone, and reduced glutathione (GSH). Moreover, the concentration of MDA, oxidized glutathione (GSSG), and proinflammatory cytokines were increased. *G. biloba* extract (EGb-761) effectively reduced oxidative stress and adverse changes in a dose-dependent manner [50]. Similarly, to reduce MTX-induced testicular damage and reproductive disorders in rats, Kamel et al. [51] used the antioxidant properties of ginseng, while Felemban et al. [52] used the ability of amygdalin (Vit B17) to inhibit lipid peroxidation and free radical scavenging. An extract from *Gardenia jasminoides* Ellis, a genipin, reversed the adverse changes in spermatogenesis and fertility caused by photoperiod disturbance in male mice. A normalization of sex hormones and proteins involved in steroidogenesis was observed, as well as a reduction of atrophy of the seminiferous tubules, decreased vacuolization, and restoration of typical control animals’ sperm motility and concentration [41].

In our study, the vast majority of the adverse effects of circadian disruption were suppressed by the sericin used in the experiment (Figs. 1-8). Sericin constitutes as much as 20–30% of the mass of silkworm cocoons and is a valuable natural protein from the textile industry by-products. Its contemporary use in medicine is related to high biological activity, lack of immunoreactivity, hydrophilicity, a tendency to form a gel, and the ability to create films or scaffolds when combined with other substances. Therefore, it is proposed to use sericin for wound healing, artificial skin, and contact lens production. Silk proteins (mainly Ala, Gly, Ser, Val, and Thr) administered to rats under exercise stress improved physical endurance but also increased testosterone level and sperm count [53]. Consumed sericin has a protective effect on the gastrointestinal tract (anti-tumor, anti-diabetic, anti-constipation properties), and due to its strong affinity to selected drugs, it is considered a drug carrier [22–24, 54–56]. Moreover, its high antioxidant potential [24, 57–59] creates new opportunities for its use wherever oxidative stress occurs, leading to a direct or indirect cause of organism dysfunction. Since reproductive dysfunction due to disturbed photoperiod is associated with increased oxidative stress, thus we postulate that sericin can be a good way of mitigating these adverse effects.

## Conclusions

Reproductive disorders caused by human activity modification and disturbed photoperiod are a contemporary problem of civilization. Our research has shown that sericin can reduce the stress caused by photoperiod disorders regarding testicular function, sex hormone levels, and sperm quantity and quality. Silk sericin is a digestible, biocompatible, and biodegradable protein, which creates a safe prospect of its use in the case of people living under the incorrect photoperiod, including the ubiquitous ALAN. Undoubtedly, further research on the prevention/treatment of male infertility using sericin is advisable before the application.

## Data Availability

All data gathered and analyzed in this study are included in this article.

## Abbreviations

AGP: Adreno-gonadal primordium
ALAN: Artificial light at night
CG: Control group
DHEAS: Dehydroepiandrosterone sulfate
GnRH; GnIH: Gonadotropin-releasing/inhibitory hormone
GSH: Reduced glutathione
GSSG: Oxidized glutathione
HPA: Hypothalamus-pituitary-adrenal
HPG: Hypothalamic-pituitary-gonadal
HRP: Horse radish peroxidase
LD: Long Day
LPG: Long-term photoperiod group
LPG-P: Long-term photoperiod group-placebo subgroup
LPG-S: Long-term photoperiod group-sericin-treated subgroup
MDA: Malondialdehyde
Mel: Melatonin
MTX: Methotrexate
PCA: Principal Component Analysis
PC1: Principal component 1
PC2: Principal component 2
PPS: Preputial separation
SD: Short day
SF-1: Steroidogenic factor-1
SCN: Suprachiasmatic nuclei
SPG: Short-term photoperiod group
SPG-P: Short-term photoperiod group-placebo subgroup
SPG-S: Short-term photoperiod group-sericin-treated subgroup
TAC: Total antioxidant capacity
WT1: Wilms’ tumor suppressor gene 1
XO: Xanthine oxidase
ZG: Zona glomerulosa
ZF: Zona fasciculate
ZR: Zona reticularis
